# Administration of a Next-Generation Probiotic *Escherichia coli* Nissle 1917-GLP-1 Alleviates Diabetes in Mice With Type 1 and Type 2 Diabetes

**DOI:** 10.1155/cjid/6675676

**Published:** 2025-01-29

**Authors:** Jie Luo, Yilin Fang, Zhanghua Qi, Fengyang Cui, Hong Hu, Shengjie Li, Tingtao Chen, Hongyan Zhang

**Affiliations:** ^1^Jiangxi Provincial Key Laboratory of Disease Prevention and Public Health, School of Public Health, Jiangxi Medical College, Nanchang University, Nanchang, China; ^2^National Engineering Research Center for Bioengineering Drugs and the Technologies, Institute of Translational Medicine, Jiangxi Medical College, Nanchang University, Nanchang, China; ^3^Jiangxi Province Key Laboratory of Bioengineering Drugs, School of Pharmacy, Jiangxi Medical College, Nanchang University, Nanchang 330031, China; ^4^Medical Center of Burn Plastic and Wound Repair, The First Affiliated Hospital, Jiangxi Medical College, Nanchang University, Nanchang, China

**Keywords:** diabetes mellitus, EcN-GLP-1, inflammation, intestinal microbiota, islet *β-*cell

## Abstract

Diabetes mellitus (DM) is a persistent and steadily progressing metabolic condition distinguished by unregulated high levels of blood glucose. GLP1 receptor agonists have recently gained recognition as first-line therapies in selected instances, as per the updated ADA guidelines, highlighting their efficacy not only in glycemic control but also in their broader health benefits. Nonetheless, the efficacy of GLP-1 is limited by its brief duration of action, rapid clearance from the body, and challenges associated with subcutaneous administration. In this study, we examined the potential diabetes-mitigating effects of a genetically engineered strain of Escherichia coli Nissle 1917 (EcN)-GLP-1, previously developed by our group. We utilized mouse models for both Type 1 diabetes mellitus (T1DM) and Type 2 diabetes mellitus (T2DM) to assess its efficacy. In the case of T1DM mice, the results revealed that EcN-GLP-1 resulted in a notable decrease in blood glucose levels. Furthermore, it exhibited a protective influence on the structural integrity of islet *β*-cells; downregulated the expressions of key inflammatory markers such as TLR-4, p-NF-*κ*B/NF-*κ*B, and Bax/Bcl-2; promoted the insulin secretion; and reinstated the perturbed diversity of microbial species to a normal state. Similarly, EcN-GLP-1 had a pronounced impact on T2DM mice, manifesting increased presence of islet *β*-cells, decreased inflammatory response and apoptosis, and regulation of lipid metabolism in the liver. In summary, the genetically modified EcN-GLP-1 strain demonstrates the ability to alleviate diabetes by enhancing the islet *β*-cell population, mitigating inflammatory reactions and apoptosis, optimizing liver lipid metabolism, and reinstating a balanced microbial diversity. These findings hold promise as a potential avenue for treating DM.

## 1. Introduction

Diabetes mellitus (DM) is a persistent metabolic disorder characterized by elevated levels of blood glucose. This increase in glucose is caused by compromised insulin production, impaired insulin function, or a combination of both factors. This condition profoundly affects the quality of life and life expectancy, as it elevates the chances of numerous complications including cardiovascular diseases, neuropathy, nephropathy, and retinopathy [1]. As per the International Diabetes Federation, diabetes affected over 537 million adults in 2021, and this figure is anticipated to surge to 783 million by 2045 [2]. Currently, diabetes management primarily revolves around lifestyle adjustments, encompassing dietary alterations, consistent physical activity, and the maintenance of a healthy weight. However, many patients require pharmacological interventions to achieve adequate glycemic control. Frequently recommended medications include metformin, sulfonylureas, meglitinides, thiazolidinediones, dipeptidyl peptidase-4 (DPP-4) inhibitors, and sodium-glucose cotransporter-2 (SGLT2) inhibitors [3]. However, these medications still have their limitations. Patients may experience hypoglycemia, weight gain, and adverse gastrointestinal effects while taking these medications [4]. Addressing these limitations and continuously improving diabetes treatments are crucial to enhance patient outcomes and lessen the impact of complications associated with this condition.

When food is consumed, the intestines produce a peptide hormone called glucagon-like peptide-1 (GLP-1) [5]. GLP-1 plays a vital role in regulating glucose levels by boosting insulin release, restraining glucagon discharge, delaying stomach emptying, and fostering a sense of fullness [6]. Due to its beneficial effects on blood glucose regulation and potential to promote weight loss, GLP-1 has emerged as a prominent subject of study in the domain of diabetes research. To manage Type 2 diabetes, drugs known as GLP-1 receptor agonists have been developed [7]. These medications mimic the action of GLP-1 and are widely used. In addition to their use in diabetes treatment, GLP-1 agonists have showed promise in managing obesity. They are approved for obesity treatment in some countries and are being studied for their potential in combination therapies with other antiobesity medications [8]. However, the native form of GLP-1 has a short duration of effectiveness because it rapidly breaks down due to the activity of the enzyme DPP-4 [9]. This necessitates the use of modified, longer acting GLP-1 agonists for therapeutic purposes, which may still have some limitations in their duration of action [10]. While GLP-1 agonists are generally well-tolerated, some patients may experience gastrointestinal adverse effects, including feelings of nausea and instances of vomiting, particularly when initiating treatment [11]. Furthermore, GLP-1 agonists are primarily administered via subcutaneous injections, which can be a barrier to patient adherence and convenience [12]. Therefore, the development of the GLP-1 oral agent is an ongoing area of research to address this limitation.

In recent years, the research has suggested that dysbiosis which is the alteration of gut microbiota, commonly known as dysbiosis, might be connected to the emergence and advancement of various metabolic conditions, including diabetes [13, 14]. There seems to be a connection between dysbiosis in gut microbiota and the deterioration of glucose metabolism and resistance to insulin [15]. Probiotics are microorganisms that are alive and, when taken in sufficient quantities, can bring about beneficial effects on an individual's health. They are commonly used to promote gut health and have been investigated for their potential in diabetes management [16, 17]. Some studies suggested that specific probiotics may improve glucose metabolism and enhance insulin sensitivity. For example, certain strains of *Lactobacillus* and *Bifidobacterium* have been found to have effects to reduce blood glucose levels and improve glycemic control [18–20]. *Escherichia coli* Nissle 1917 (EcN) is a harmless variant of *E. coli*, which has been extensively studied for its probiotic properties and potential health benefits [21]. Some studies have suggested that EcN may exert a positive impact on regulating blood sugar levels in individuals with diabetes. It is believed that the adjustment of gut microbiota by EcN could positively influence glucose metabolism and insulin sensitivity [22, 23]. In addition, EcN's ability to regulate the immune response, decrease inflammation [24], fortify gut barrier integrity [25], and impact positively on lipid metabolism [26] may be beneficial in diabetes management. Thus, EcN presents itself as a promising choice for both preventing and managing diabetes.

In the context of diabetes, engineering bacteria to produce therapeutic molecules or modulate the gut microbiota holds promise as a novel approach to diabetes management. In our previous studies, we constructed some genetically engineered strains based on a single-plasmid genome editing system within lactic acid bacteria to continuously produce GLP-1, which demonstrated positive therapeutic outcomes in obesity mice, neuropsychiatric disease, T2DM mice, and even in T2DM monkeys [27–29]. However, plasmid-engineered bacteria do have some limitations that need to be considered when utilizing them in practical settings, such as stability and loss of plasmids, low transformation efficiency, and genetic instability. Considering these problems, we proceeded to engineer a recombinant integrant *E. coli* Nissle strain for delivering GLP-1. This strain exhibited significant neuroprotective effects in mice with Parkinson's disease (PD) by reducing inflammation and addressing the gut microbiota imbalance associated with the condition [30]. In this research, we utilized the engineered EcN-GLP-1 strain in mice with both T1DM and T2DM to assess its therapeutic efficacy and the underlying mechanisms. This study offers essential experimental data for the advancement of engineered bacterial therapies and their potential translation into clinical applications.

## 2. Materials and Methods

### 2.1. Animals

To create the T1DM mouse model, 8-week-old male wild-type C57BL/6 mice (SJA Laboratory Animal Co., Ltd., China) were kept in conditions free from pathogens, with carefully controlled environmental parameters. They were subjected to a 12-h cycle of light and darkness and had unlimited access to standard laboratory food and water, while maintaining humidity at 50 ± 15% and temperature at 22 ± 2°C. After a one-week acclimation period, several measures were taken to minimize the impact of cages on the microbiome. The mice were individually housed in separate cages and provided with identical diets and sterile bedding. The researchers followed strict hygiene protocols and changed their gloves regularly while handling the cages. To induce T1DM, mice received intraperitoneal injections of streptozotocin (STZ) at a dosage of 50 mg/kg/day for five consecutive days until their blood glucose levels reached 11.1 mM (199.8 mg/dL) or higher and remained stable for 7 days [31–34]. Following this, the mice were categorized into four groups: (1) M group: This consisted of the T1DM model group, which received a daily intragastric administration of 100 *μ*L of the bacterial coating solution for 9 weeks (*n* = 12); (2) EcN group: This comprised T1DM mice that were intraperitoneally injected with 100 *μ*L of EcN suspended in the coating fluid, with a concentration of 1010 CFU/mL(*n* = 12); (3) EcN-G group: This included T1DM mice that received intraperitoneally injected with 100 *μ*L of EcN-GLP-1 suspended in the coating fluid, with a concentration of 1010 CFU/mL(*n* = 12); (4) P group: This consisted of T1DM mice that administered the GLP-1 receptor agonist exenatide via intraperitoneal injection at a dose of 24 nmol/kg/day (*n* = 12). All mice received their respective treatments once daily for a duration of 9 weeks. In addition, 15 more wild-type C57BL/6 mice were included as the control group (referred to as the C group).

For the T2DM mouse model, 8-week-old male wild-type C57BL/6 mice were given a period of 1 week for acclimatization. Following the acclimation period, they were fed a high-fat diet (comprising 20% protein, 20% carbohydrates, 60% fat, and providing 5.24 kcal/g; obtained from Xietong Bio, China, XTHF60) for a total of 9 weeks. Out of the mice, 12 were chosen at random to form the control group (C group, *n* = 12), and they received the bacterial coating solution intragastrically in a volume of 100 *μ*L. The rest of the mice were induced into T2DM using intraperitoneal injection of STZ at a dose of 30 mg/kg, which was dissolved in a citrate buffer at a concentration of 0.1 mol/L and had a pH of 4.5. This injection of STZ was administered over five consecutive days. Mice exhibiting fasting blood glucose levels exceeding 11.1 mmol/L were included in this research and were then randomly allocated into four distinct groups: M group, EcN group, EcN-G group, and P group. All administration methods and solutions used in these experiments were identical to those for the experiments conducted with T1DM mice.

In each group of mice, blood glucose levels were assessed weekly, and body weight was recorded on a weekly basis. At Week 9, an analysis of the composition of the fecal microbiota was conducted. In addition, seven mice were selected for sacrifice: four for the purpose of conducting western blot analysis on the pancreas, while the remaining three were designated for hematoxylin and eosin (HE) staining and immunohistochemical staining of the pancreas. All experimental procedures involving animals adhered to the Guidelines for Care and Use of Laboratory Animals set forth by Nanchang University. Approval for the protocols was obtained from the Animal Ethics Committee of Nanchang Royo Biotechnology Co., Ltd, Nanchang, China (approval number: RYE2019100801).

### 2.2. Glucose Tolerance Test (GTT)

Before each test, the mice underwent a 12-h fasting period, and their blood glucose were measured at the starting point (time = 0). The GTTs involved administering an intraperitoneal injection of glucose at a dosage of 1.5 mg/g to the mice. Blood glucose levels were then measured at 30-, 60-, 90-, and 120-min intervals postinjection.

### 2.3. High-Throughput Sequencing Analyses

During the experiment, Parsenor Bio (China) received the fecal samples from the mice for analysis of 16S rDNA using high-throughput sequencing. The bacterial genomic DNA was extracted and analyzed using the 16S rRNA V3-V4 sequencing method with 338 F/806R primers. The primers used were 338F: 5′-ACTCCTACGGGAGGCAGCAG-3′ and 806R: 5′-GGACTACHVGGGTWTCTAAT-3′, where H represents A/T/C, V represents G/A/C, and W represents A/T. The high-throughput sequencing provided raw data, and the paired ends of the raw DNA fragments were processed using Cutadapt software and the UCHIME algorithm. Afterward, the UPARSE software package was utilized for sequence analysis. Sequences with a similarity of ≥ 97% were grouped into the same operational taxonomic unit (OTU). The number of reads corresponding to each OTU in each sample was recorded. To ensure alpha diversity analysis, samples were randomly selected to match the lowest sequence number of the corresponding OTU. The most prevalent and characteristic sequences from each OTU were isolated, and they were then compared to the RDP database for classification, resulting in a species abundance table for further analysis. To ensure accessibility, the high-throughput sequencing data have been submitted to NCBI. The corresponding GenBank accession number is provided as PRJNA1016258.

### 2.4. Pathological Histology

To preserve the pancreatic tissue, it was initially fixed in a solution of 4% paraformaldehyde. Subsequently, it underwent a series of steps, including dehydration in ascending concentrations of alcohol, clearing in xylene, and embedding in paraffin for further analysis. The paraffin-embedded tissues were then sliced into sections with a thickness of 5 μm. These sections were then rehydrated through a sequence that involved xylene and graded alcohols. HE staining was conducted on the sections for a duration of 1–5 min. After staining, the sections underwent rinsing, dehydration using graded alcohol, transparency achieved using xylene, and were then firmly secured using neutral resin. In the process of immunofluorescence analysis, antigen retrieval was conducted following rehydration, and the sections were probed with an anti-insulin antibody (#4590S) bought from cell signaling technology. In the case of oil red O staining, the frozen tissue sections were first washed with a PBS buffer. They were then exposed to oil red O solution for a duration of 10–20 min. After washing, the tissues were counterstained with hematoxylin for a duration 5 min and subsequently mounted with glycerol after an additional wash.

### 2.5. Western Blot Analysis

The pancreatic tissue was homogenized and then was lysed using a radioimmunoprecipitation assay (RIPA) lysis buffer. The resulting lysates were centrifuged to collect the supernatant (12 000 g, 10 min, 4°C). The protein concentration in the supernatant was determined using a BCA kit (Thermo Fisher Scientific, A53226). Subsequently, the proteins underwent gel electrophoresis (SDS-PAGE) using a 10%–12% gel and were subsequently transferred onto polyvinylidene fluoride (PVDF) membranes. To block the PVDF membranes, they were incubated with 5% milk Tris-buffered saline with Tween 20 (TBST) for a period of 1 h at room temperature. Overnight incubation at 4°C with appropriately diluted primary antibodies followed the exposure of PVDF membranes to primary antibodies. After washing the membranes with TBST, a secondary antibody diluted in 1% milk TBST was added and incubated for 1 h at room temperature. The primary antibodies employed in this study were as follows: anti-TLR4 (1:1000; #sc-293072) was purchased from Santa Cruz Biotechnology, anti-p-NF-*κ*B (1:1000; #ab86299) was bought from Abcam, and other antibodies include antimyeloid differentiation factor 88 (MyD88) (1:1000; #4283), anti-nuclear factor kappa-B (NF-*κ*B) (1:1000; #8242S), anti-*β*-actin (1:1000; #4970S), anti-B-cell lymphoma-2 (Bcl-2, 1:1000; #3498S), and anti-Bcl-2 associated X protein (Bax, 1:1000; #14796S) were obtained from cell signaling technology. The immune complexes bound to the membrane were visualized using an enhanced chemiluminescence system from Thermo Fisher Scientific, the United States of America. Densitometric analysis was performed using ImageJ software developed by NIH for quantification purposes.

### 2.6. Statistical Analysis

GraphPad Prism version 7.0, developed by GraphPad Software, Inc. (the United States of America), was utilized for the processing, analysis, and generation of graphical representations. The mean ± standard deviation (SD) is used to present the results. Significance testing was performed through either one-way or two-way ANOVA, followed by post hoc analysis using Tukey's test. A *p* value lower than 0.05 was considered statistically significant in accordance with the established international conventions.

## 3. Results

### 3.1. The Beneficial Impacts of EcN-GLP-1 on Blood Glucose Regulation and Pancreatic Inflammation in T1DM Mice

**Figure 1:**
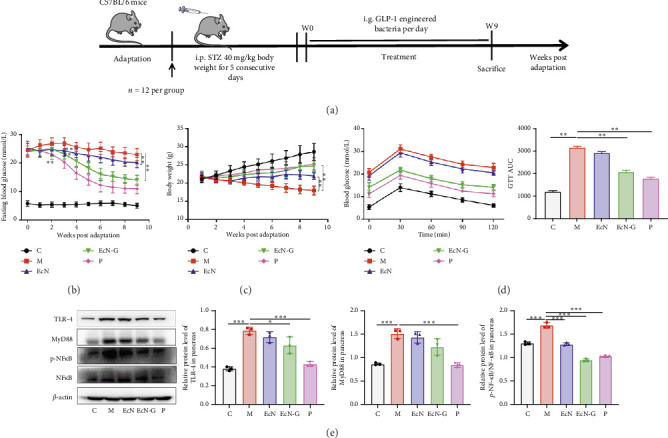
The engineered strain EcN-GLP-1 significantly alleviates diabetic symptoms and pancreatic inflammation in T1DM mice. (a) Scheme of animal experiment. (b) Fasting blood glucose levels tested from mice tail veins. (c) Body weight. (d) Blood glucose levels and the AUC in the GTT test. (e) The western blot results indicated that the expression levels of proinflammatory proteins including TLR-4, MyD 88, and the ratio of p-NF-*κ*B/NF-*κ*B in pancreas of the engineered strain–treated mice were significantly reduced. C group: wild-type C57BL/6 mice (*n* = 12); M group: T1DM mice (*n* = 12); EcN group: *E.coli* Nissle 1917–treated T1DM mice (*n* = 12); EcN-G group: EcN-GLP-1–treated T1DM mice (*n* = 12); P group: exenatide-treated T1DM mice (*n* = 12). Data are presented as mean ± SD. ⁣^∗^*p* < 0.05 and ⁣^∗∗^*p* < 0.01.

An evaluation of the therapeutic effects of EcN-GLP-1 was conducted using a mouse model for T1DM. Five groups of T1DM mice, including a normal control group (C group), T1DM model group (M group), EcN group, EcN-G group, and P group, were categorized for experimentation (Figure 1(a)).  Figure 1(b) demonstrates a significant increase in the levels of fasting blood glucose in T1DM mice, exceeding 24.5 mmol/L, yet the control group maintained stable or normal levels. By the third week, mice that received treated with EcN-GLP-1 or EcN both exhibited noteworthy reduction in blood glucose levels compared to the untreated T1DM mice (23.2 or 24.3 mmol/L versus 26.9 mmol/L, *p* < 0.01). In contrast, the group treated with the positive drug exenatide exhibited improvement as early as the second week (23.1 mmol/L versus 26.7 mmol/L, *p* < 0.01). Weight loss is a common occurrence in T1DM mice, but our results showed that EcN-GLP-1 could inhibit this weight loss, akin to the impact of exenatide (Figure 1(c)). Furthermore, results from the GTT and analysis of the area under the glucose curve (AUC), where both the treatments with EcN-GLP-1 (2076 versus 3155, *p* < 0.01) and exenatide (1769 versus 3155, *p* < 0.01) showed markedly reduced AUC values compared to the untreated T1DM mice (Figure 1(d)), both indicated that EcN-GLP-1 improved blood glucose tolerance in mice with T1DM.

Having established a strong association between pancreatic inflammation and the damage to islet *β*-cells, we proceeded to examine the impact of EcN-GLP-1 on the inflammatory signaling pathway. Our findings demonstrated that the genetically modified bacteria, similar to exenatide, resulted in a notable decrease in the expression of TLR-4 (0.79 versus 0.63 and 0.43) and p-NF-*κ*B/NF-*κ*B (1.69 versus 0.94 and 1.02), when compared to the expression levels observed in T1DM mice (Figure 1(e); *p* < 0.01). Unlike exenatide, EcN-GLP-1 showed no obvious effect to the expression of MyD88 (1.51 versus 1.23, *p* > 0.05).

### 3.2. The Protective Impact of EcN-GLP-1 on Pancreatic Islets in T1DM Mice

**Figure 2:**
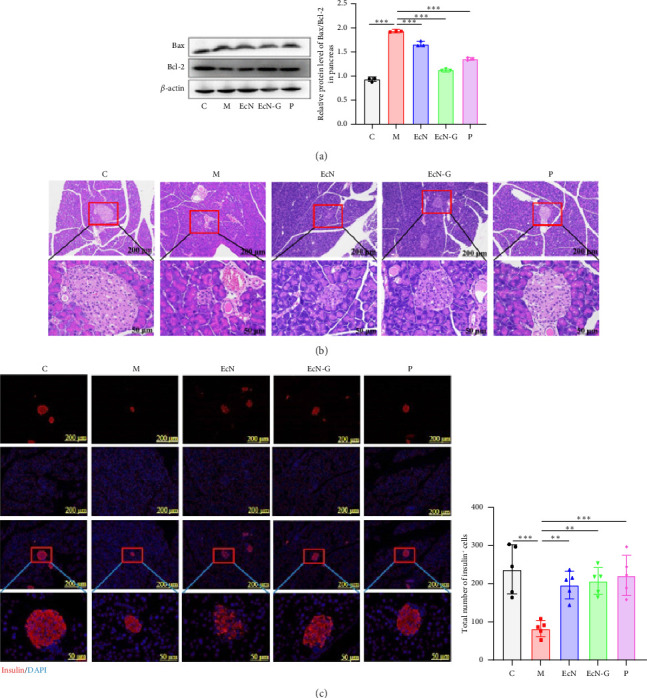
The engineered strain EcN-GLP-1 could reduce pancreas injury in T1DM mice. (a) Western blotting results of the expression ratio of Bax/Bcl-2. (b) The HE staining of the pancreas. The images in the second line are the magnified images of the red square above (magnification: × 100 and × 400, scale bar = 200 μm and 50 μm). (c) The immunofluorescence analysis of insulin in the pancreas. The images in the fourth line are the magnified images of the red square above (magnification: × 100 and × 400, scale bar = 200 μm and 50 μm). M: T1DM mice (*n* = 12); EcN group: *E.coli* Nissle 1917–treated T1DM mice (*n* = 12); EcN-G group: EcN-GLP-1–treated T1DM mice (*n* = 12); P group: exenatide-treated T1DM mice (*n* = 12). Data are presented as mean ± SD. ⁣^∗^*p* < 0.05 and ⁣^∗∗^*p* < 0.01.

To assess the impact of EcN-GLP-1 on the pancreas of mice with T1DM, we examined crucial proteins related to apoptosis. When compared to the T1DM mouse group, all three treatments involving EcN, EcN-GLP-1, and exenatide resulted in a substantial decrease in the Bax/Bcl-2 ratio (1.95 versus 1.67, 1.12 and 1.35, *p* < 0.01) (Figure 2(a)), suggesting that the probiotic strain and its engineered bacteria both could effectively inhibit pancreatic cells apoptosis. In addition, significant variations in both the quantity and pattern of islet cells were observed through histological examination of pancreas tissues (Figure 2(b)). In T1DM mice, the islet cells showed severe shrinkage, and there was a significant decrease in both size and number of islet cell clusters. However, the administration of either EcN-GLP-1 or exenatide effectively suppressed apoptosis of islet cells and facilitated their repair. Likewise, insulin immunofluorescence staining for insulin in pancreatic tissue sections revealed a significant decrease in insulin-secreting *β*-cells in T1DM mice compared to the control group (Figure 2(c)). Conversely, the area occupied by insulin-positive cells was notably larger in mice that received treatment with the genetically modified bacteria, indicating that EcN-GLP-1 could stimulate the proliferation of pancreatic *β*-cells and increase levels of insulin expression.

### 3.3. The Modulatory Impact of EcN-GLP-1 on the Gut Microbiota of T1DM Mice

**Figure 3:**
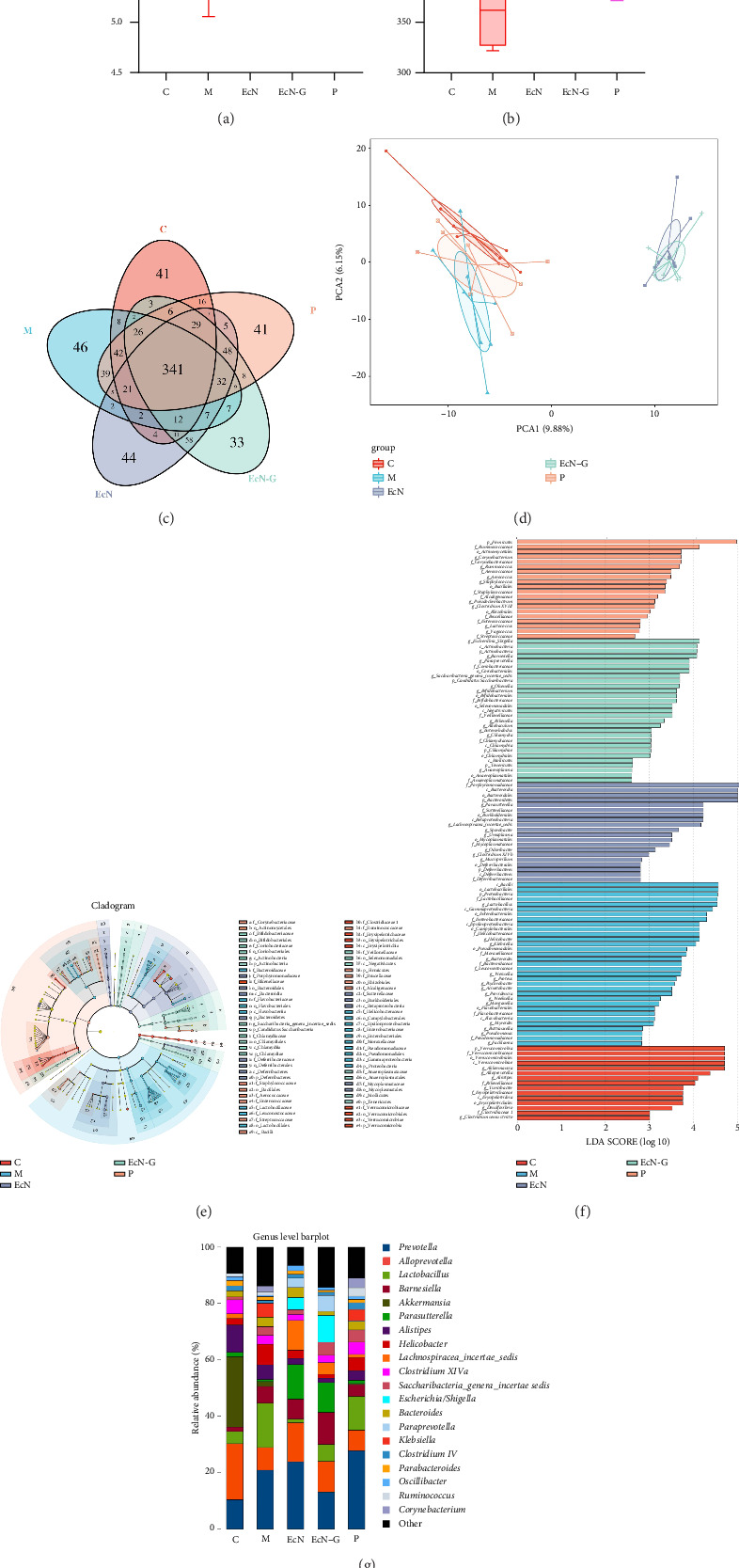
The engineered strain EcN-GLP-1 restores the intestinal microbiota in T1DM mice. The engineered strain increased the Shannon (a) and Chao 1 (b) indices of the gut microbiota. (c) The total number of core operational taxonomic units (OTUs) shared and that are unique in the Venn diagram. (d) PcoA analysis of the samples of the different groups. (e) LEfSe cladogram. (f) LEfSe analysis with linear discriminant analysis. (g) Barplots of the relative abundance of gut bacteria at the genus level. C: normal mice (*n* = 12); M: T1DM mice (*n* = 12); EcN group: *E.coli* Nissle 1917–treated T1DM mice (*n* = 12); EcN-G group: EcN-GLP-1–treated T1DM mice (*n* = 12); P group: exenatide-treated T1DM mice (*n* = 12). Data are presented as mean ± SD. ⁣^∗^*p* < 0.05 and ⁣^∗∗^*p* < 0.01.

To delve deeper into the connection between EcN-GLP-1, T1DM, and the gut microbiota, we gathered fecal samples from each mouse group and performed high-throughput sequencing to analyze the intestinal microbiota. We obtained a total of 1,432,772 valid tags and identified 14,433 OTUs, averaging 2886.6 per group. The five groups were assessed for alpha diversity, encompassing community composition and diversity (which includes richness and evenness). As depicted in Figures 3(a) and 3(b), both diversity (measured by the Shannon's index) and bacterial richness (measured by the Chao1 index), were notably decreased in T1DM mice. However, treatment with EcN, EcN-GLP-1, and exenatide resulted in significant increases in both diversity and bacterial richness in the intestinal microbiota. Furthermore, the analysis of the Venn diagram demonstrated that there was a total of 341 OTUs that were common to all five groups. In addition, each group had unique OTUs, with 41 in the control mice, 46 in the T1DM mice, 44 in the EcN-treated mice, 33 in the EcN-GLP-1–treated mice, and 41 in the exenatide-treated mice (Figure 3(c)). Moreover, principal component analysis (PCA) revealed that the control group had clustered data points, while the T1DM group had more dispersed data points. Significantly, the gut microbiota community composition in mice receiving EcN and EcN-GLP-1 treatments showed significant similarity, while it differed markedly from the microbiota patterns in the control group, T1DM mice, and mice-treated with exenatide (Figure 3(d)).

The application of linear discriminant analysis effect size (LEfSe) examination unveiled notable distinctions in microbiome composition across the five groups at different levels of taxonomy. To evaluate the varying influence of each community on the phylogenetic allocation, we used an LDA cutoff of 2.5. In total, 71 taxa were detected among these five groups (Figure 3(e)). The most influential taxa, at the genus level, that contributed to the disparities were presented (Figure 3(f)). Specifically, at the levels of genus, variations in 20 genera with the highest occurrence among the five groups were observed (Figure 3(g)). We selected several representative bacteria that are closely linked to diabetes for more detailed analysis. As depicted in the figure, the control mice displayed the greatest relative abundance of *Alloprevotella*. Conversely, there was a significant rise in the presence of intestinal microorganisms, including *Helicobacter*, *Bacteroides*, and *Lactobacillus* (*p* < 0.05), in T1DM mice. Comparatively, treatment with EcN-GLP-1 led to an increase in *Alloprevotella*, *Escherichia*, and *Parasutterella* (*p* < 0.05), along with a decrease in the levels of *Helicobacter*, *Bacteroides*, and *Lactobacillus* (*p* < 0.05) when compared to the T1DM group. Likewise, exenatide treatment led to an increase in *Alloprevotella* (*p* < 0.05) and a decrease in *Bacteroides* and *Helicobacter* (*p* < 0.05) (Figure 3(g) and Supporting Figure 1a).

### 3.4. The Treatment and Protective Effects of EcN-GLP-1 on the T2DM Mice and the Pancreas

**Figure 4:**
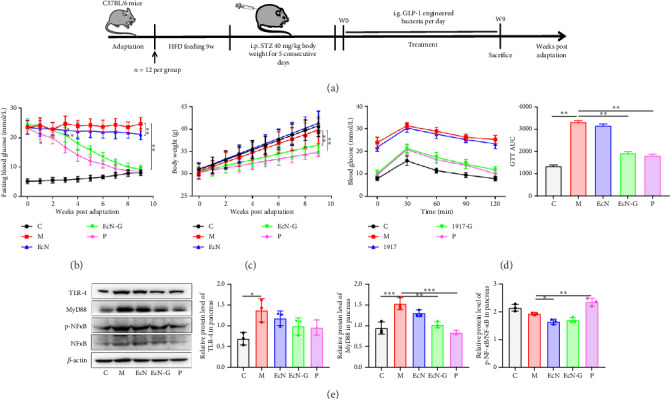
The improvement effects of EcN-GLP-1 on diabetic symptoms and pancreatic inflammation in T2DM mice. (a) Scheme of the animal experiment. (b) Fasting blood glucose levels. (c) Body weight. (d) Blood glucose levels and the AUC in the GTT test. (e) The western blot results of the expression level of proinflammatory proteins including TLR-4, MyD 88, and the ratio of p-NF-*κ*B/NF-*κ*B in the pancreas. C group: wild-type C57BL/6 mice (*n* = 12); M group: T2DM mice (*n* = 12); EcN group: *E.coli* Nissle 1917–treated T2DM mice (*n* = 12); EcN-G group: EcN-GLP-1–treated T2DM mice (*n* = 12); P group: exenatide-treated T2DM mice (*n* = 12). Data are presented as mean ± SD. ⁣^∗^*p* < 0.05 and ⁣^∗∗^*p* < 0.01.

In order to investigate the potential therapeutic effects of the engineered strain EcN-GLP-1, a mouse model of T2DM was used. The mice were divided into five groups: the control group (referred to as C group), the T2DM model group (referred to as M group), the EcN group, the EcN-G group, and the P group (refer to Figure 4(a)). Subsequent tests were conducted to evaluate the influence of the engineered bacteria EcN-GLP-1 on body weight, blood glucose levels, and glucose tolerance in T2DM mice. Starting from the third week, the levels of fasting blood glucose in mice receiving EcN-GLP-1 treatment showed a decreasing trend and were notably lower compared to the M group (21.0 mmol/L versus 24.7 mmol/L, *p* < 0.01) (Figure 4(b)). In contrast to T1DM mice, where weight increased as feeding time increased, the body weight of T2DM mice increased progressively. However, treatment with EcN-GLP-1 significantly reduced the weight gain symptoms in T2DM mice, similar to the effect of exenatide (Figure 4(c)). The findings from the GTT and AUC analyses in T2DM mice were consistent with those observed in T1DM mice. EcN-GLP-1 notably enhanced blood glucose tolerance and decreased the AUC value (*p* < 0.01) (Figure 4(d)). In addition, the expression of MyD88 was notably decreased by both the engineered bacteria and exenatide (1.54 versus 1.03 and 0.84, *p* < 0.01), while EcN-GLP-1 did not increase the expression of p-NF*κ*B/NF*κ*B (1.93 versus 1.70, *p* > 0.05) as exenatide do (1.93 versus 2.35, *p* < 0.01), and none treatments had an obvious effect on the expression of TLR-4 compared to the T2DM mice (1.38 versus 0.99 and 0.96, *p* > 0.05) (Figure 4(e)).

**Figure 5:**
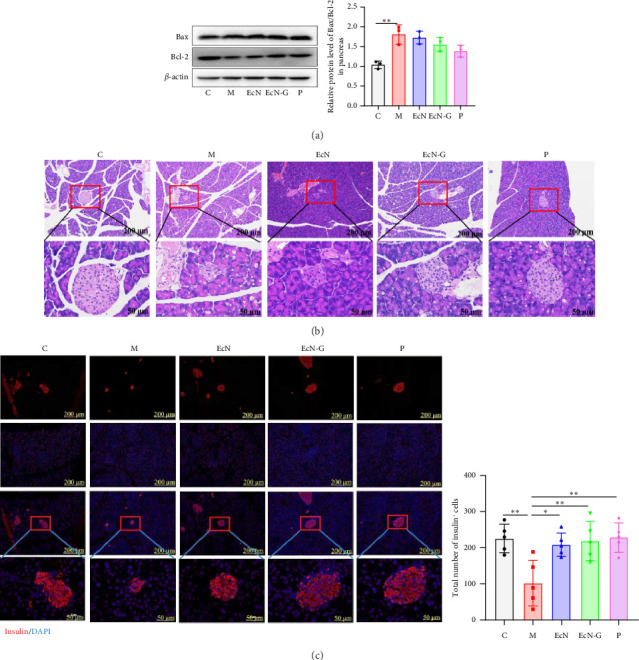
The engineered strain EcN-GLP-1 could reduce pancreas injury in T2DM mice. (a) Western blotting results of the expression ratio of Bax/Bcl-2. (b) The HE staining of the pancreas. The images in the second line are the magnified images of the red square above (magnification: × 100 and × 400, scale bar = 200 μm and 50 μm). (c) The immunofluorescence analysis of insulin in the pancreas. The images in the fourth line are the magnified images of the red square above (magnification: × 100 and × 400, scale bar = 200 μm and 50 μm). C group: wild-type C57BL/6 mice (*n* = 12); M group: T2DM mice (*n* = 12); EcN group: *E.coli* Nissle 1917–treated T2DM mice (*n* = 12); EcN-G group: EcN-GLP-1–treated T2DM mice (*n* = 12); P group: exenatide-treated T2DM mice (*n* = 12). Data are presented as mean ± SD. ⁣^∗^*p* < 0.05 and ⁣^∗∗^*p* < 0.01.

The outcomes observed in the pancreatic islets of T2DM mice with EcN-GLP-1 were in line with the anticipated effects. When compared to the M group, both EcN-GLP-1 and exenatide resulted in a significant reduction in the Bax/Bcl-2 ratio (1.81 versus 1.55 and 1.38, *p* > 0.05) (Figure 5(a)). This indicates that they may not have the same impact on pancreatic cell apoptosis in T2DM mice as observed in T1DM mice. However, EcN-GLP-1 treatment notably facilitated the regeneration of islet cells (Figure 5(b)) and increased the count of insulin-positive cells, indicating the promotion of pancreatic *β*-cell proliferation and an increase in insulin expression (Figure 5(c)). While EcN-GLP-1 may not inhibit pancreatic cell apoptosis, other findings underscore its favorable effects on enhancing pancreatic function and insulin production in T2DM mice.

### 3.5. The Regulatory Influence of EcN-GLP-1 on the Gut Microbiota and Liver Lipid of T2DM Mice

**Figure 6:**
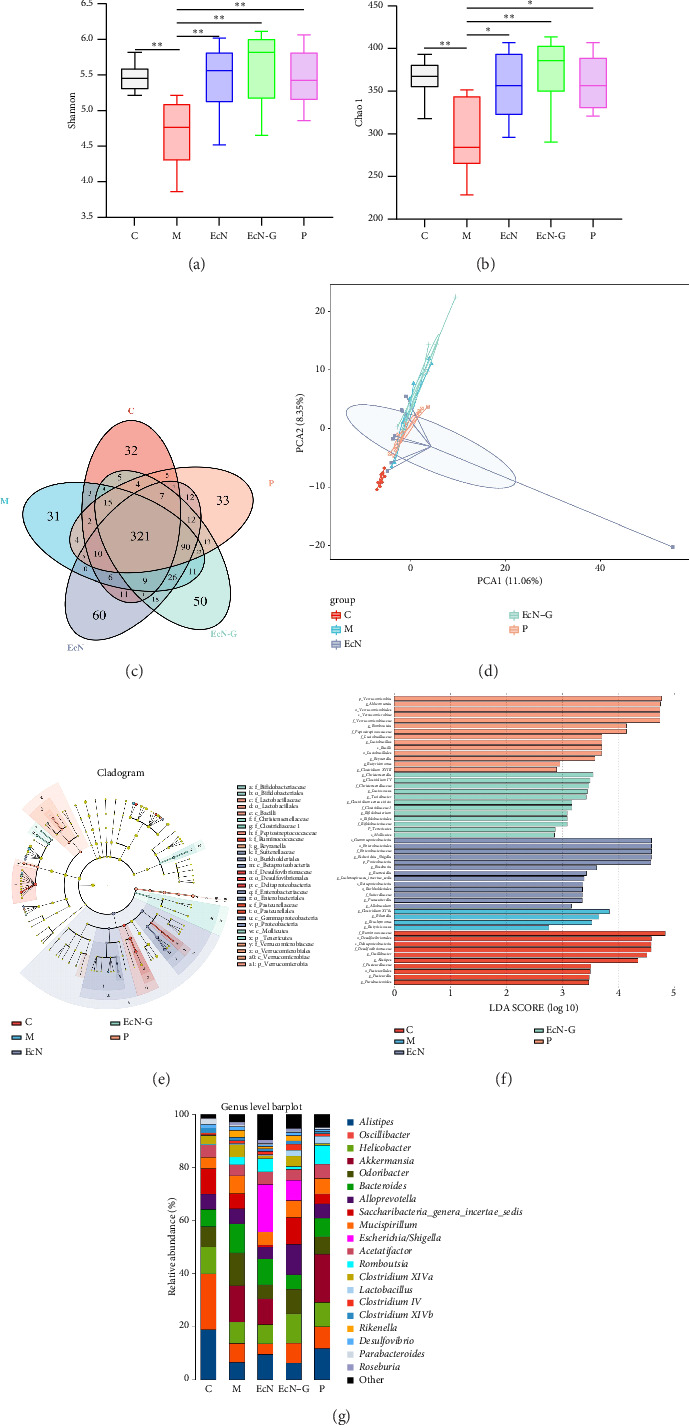
The engineered strain EcN-GLP-1 restores the gut mucosal barrier and intestinal microbiota in T2DM mice. The engineered strain increased the Shannon (a) and Chao 1 (b) indices of the gut microbiota. (c) The total number of core operational taxonomic units (OTUs) shared and that are unique in the Venn diagram. (d) PcoA analysis of the samples of the different groups. (e) LEfSe cladogram. (f) LEfSe analysis with linear discriminant analysis. (g) Barplots of the relative abundance of gut bacteria at the genus level. C: normal mice (*n* = 12); M: T2DM mice (*n* = 12); EcN group: *E.coli* Nissle 1917–treated T2DM mice (*n* = 12); EcN-G group: EcN-GLP-1–treated T2DM mice (*n* = 12); P group: exenatide-treated T2DM mice (*n* = 12). Data are presented as mean ± SD. ⁣^∗^*p* < 0.05 and ⁣^∗∗^*p* < 0.01.

Consistent with the analysis of high-throughput sequencing conducted in T1DM mice, both the diversity and bacterial richness were significantly increased indicating that EcN-GLP-1 had the potential to normalize the gut microbiota in T2DM mice (Figures 6(a) and 6(b)). The utilization of a Venn diagram showed that there were 321 shared OTUs among all groups, with each group also having a number of unique OTUs that were 32, 31, 60, 50, and 33, respectively (Figure 6(c)). The PCoA result showed that there was a close similarity between the samples from the control group and the mice treated with EcN-GLP-1 (Figure 6(d)). In the LEfSe analysis, 28 taxa were identified across these five groups (Figures 6(e) and 6(f)), and at the genus level, *Alistipes* displayed the most abundance among the control mice. Similarly, treatment with EcN-GLP-1 led to an increase in *Escherichia* (*p* < 0.01) and while decreasing the abundance of *Bacteroides* (*p* < 0.01) in T2DM mice. But unlike in T1DM mice, EcN-GLP-1 treatment also resulted in an increase in *Clostridium IV* (*p* < 0.01) and a decrease in *Alistipes* and *Rikenella* (*p* < 0.05) (Figure 6(g) and Supporting Figure 1b).

**Figure 7:**
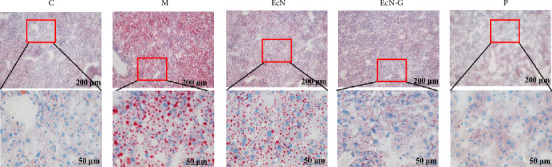
The regulation of EcN-pMG36e-GLP-1 on the liver lipid metabolism in T2DM mice. The oil red staining of the liver. The images in the second line are the magnified images of the red square above (magnification: × 100 and × 400, scale bar = 200 μm and 50 μm). C group: wild-type C57BL/6 mice (*n* = 12); M group: T2DM mice (*n* = 12); EcN group: *E.coli* Nissle 1917–treated T2DM mice (*n* = 12); EcN-G group: EcN-GLP-1–treated T2DM mice (*n* = 12); P group: exenatide-treated T2DM mice (*n* = 12). Data are presented as mean ± SD. ⁣^∗^*p* < 0.05 and ⁣^∗∗^*p* < 0.01.

Furthermore, given the elevated incidence of fatty liver in T2DM mice, we assessed the impact of EcN-GLP-1 on liver of mice. As depicted in Figure 7, liver oil red O staining in T2DM mice revealed a substantial accumulation of crimson lipid droplets. Nonetheless, both EcN-GLP-1 and exenatide treatments substantially decreased the lipid droplet region and mitigated hepatic fat accumulation.

## 4. Discussion

DM is a chronic condition that affects the metabolism and is caused by a combination of genetic and environmental factors [35]. Despite ongoing efforts, the range of preventive and therapeutic approaches remains constrained. Challenges such as drug resistance, undesirable side effects, discomfort in administration, and substantial financial burden contribute to adverse impacts on treatment outcomes and the well-being of patients [36]. It is worth mentioning that GLP-1 and its analogs have demonstrated potential as effective incretin agents, which can be used to manage T1DM in overweight or obese adults [37]. Nevertheless, the brief duration of GLP-1's biological activity poses a hurdle, necessitating weekly subcutaneous injections. The previous research endeavors have endeavored to develop oral administration methods, often leveraging Lactobacilli [28, 29]. Acknowledging the limitations of plasmid-modified bacteria, we have developed a novel recombinant EcN strain to carry and deliver GLP-1. This innovative approach has been tested in both T1DM and T2DM mice to assess its therapeutic efficacy and potential underlying mechanisms in this study.

The probiotic strain EcN has demonstrated encouraging progress in its potential application for diabetes management [38]. Research in this field suggests that EcN holds promise for individuals with diabetes due to its capacity to positively influence gut microbiota, mitigate inflammation, attenuate autoimmune responses, and directly impact glucose regulation. In addition, EcN has been explored as a vehicle for oral delivery of bioactive substances [39]. This pathway facilitates the organism's natural journey through the GI tract to the intestines, where it interfaces with gut lining and, ideally, positively influences metabolic processes pertinent to GLP-1 therapy, such as insulin regulation and glucose balance. Investigations have delved into incorporating therapeutic payloads, encompassing proteins, peptides, and nucleic acids, into the EcN framework. This strategy enables precise release and delivery at specific gut locations, potentially yielding targeted and sustained therapeutic outcomes [40–42]. In contrast to conventional plasmid-engineered bacteria, harnessing EcN as a chromosomal engineered bacterium offers distinct advantages, particularly in terms of stability, safety, targeted delivery, and controlled expression. These attributes position EcN as a promising platform for diverse biotechnological and therapeutic applications, especially when necessitating enduring, dependable, and site-specific genetic modifications [43]. In a prior study, we successfully engineered the strain EcN-GLP-1, demonstrating its resilience to the harsh conditions of the intestinal environment and its strong colonization in the gastrointestinal tract. This translated into noteworthy probiotic effects and consistent GLP-1 expression [30]. Continuous exploration and refinement are vital to fully harness these capabilities safely and effectively. Building on this foundation, we further investigated the impact of utilizing EcN-GLP-1 in both T1DM and T2DM mouse models. Our findings indicated that this engineered strain effectively lowered blood glucose levels, regulated body weight, and enhanced glucose tolerance in both T1DM and T2DM mice (Figures 1 and 5). These effects were consistent with those observed with the hypoglycemic drug exenatide, which was among the first GLP-1 receptor agonists approved for clinical use and has a substantial body of research behind it, including pharmacokinetic, pharmacodynamic, and clinical trial data.

Despite distinct etiologies and pathological characteristics between T1DM and T2DM, they do share common traits, including the irreversible deterioration of islet *β*-cells [44, 45]. Hence, a medication that stimulates the proliferation of islet *β*-cells could potentially deter the onset of both T1DM and T2DM at their root. GLP-1 has been proven to actively foster the growth and survival of pancreatic islet *β*-cells. It effectively counteracts apoptotic processes that contribute to the reduction in *β*-cell mass, thereby aiding the preservation of functional *β*-cell populations [46]. Furthermore, our preceding investigation revealed notable outcomes. By employing *L. plantarum* -pMG36e-GLP-1 in mice with T2DM, the morphology and structure of *β*-cell were restored and the expression of proteins involved in cell death regulation was modulated [47]. This underscores the potential antidiabetic efficacy of GLP-1, and the GLP-1-expressing engineered strain in the context of diabetes management.

To begin, a mouse model of T1DM was induced by STZ. STZ exposure results in an elevation in the levels of reactive oxygen species (ROS), which accelerates DNA damage in pancreatic islet *β*-cells and leads to their direct destruction, thus mimicking the characteristic symptoms of human T1DM [48]. Islet *β*-cell dysfunction primarily occurs in the context of inflammation affecting islet cells. The TLR-4/NF-*κ*B inflammatory pathway, known to promote diabetic processes, exhibits heightened expression, ultimately culminating in substantial irreversible damage to a significant portion of islet *β*-cells [49]. Consistent with findings from mice treated with exenatide, the administration of EcN-GLP-1 led to a significant reduction in blood glucose levels. Moreover, it exhibited a protective effect on the morphology and density of islet *β*-cells. Notably, the levels of TLR-4 and p-NF*κ*B/NF-*κ*B experienced a decrease, with the exception of MyD88, as depicted in Figure 2. Considering that abnormal apoptosis of islet *β*-cells characterizes T1DM, we proceeded to evaluate the expressions of Bax/Bcl-2 in pancreatic tissue. Both Bax and Bcl-2, as part of the Bcl-2 family, play opposing roles in the regulation of apoptosis. Bax undergoes upregulation during apoptosis, whereas Bcl-2 serves as a critical antiapoptotic protein. The ratio of Bax/Bcl-2 commonly evaluates their combined influence [50]. Complementing the notably expanded region of insulin-positive cells confirmed via immunofluorescence analysis, these results collectively suggest that EcN-GLP-1 may restore the quantity and functionality of islet *β*-cells in T1DM mice by mitigating the Bax/Bcl-2 ratio and stimulating insulin secretion (Figure 2).

An increasing amount of research suggested that dysbiosis of the intestinal microbiota composition may be linked with a heightened risk of DM. Shifts in the relative abundance of specific microbial taxa, such as decreased diversity, have been associated with T1DM development. In this study, both types of DM mice exhibited lower diversity in their gut microbiota compared to the normal mice, indicating an imbalance within the microbial ecosystem in the intestines. Nevertheless, administration of EcN-GLP-1 increased both the diversity and abundance of the gut microbiota, facilitating the restoration of intestinal homeostatic equilibrium. Remarkably, the three distinct groups of treatments displayed similar yet distinct microbiota profiles, potentially influenced by the administration of probiotics or hypoglycemic drugs (Figures 3 and 6). Furthermore, in both types of DM mice, the engineered bacterium EcN-GLP-1 demonstrated the ability to decrease the relative abundance of *Bacteroides* while increasing the levels of *Escherichia*. *Bacteroides* exhibited higher prevalence in T1DM individuals compared to healthy subjects [51]. The increased *Escherichia* is due to the administration of EcN, which is a probiotic bacterium without any known toxins. Furthermore, EcN-GLP-1 raised the abundance of *Alloprevotella* in T1DM mice while lowered *Rikenella* in T2DM mice. *Alloprevotella* is known as a producer of short-chain fatty acids (SCFAs), often exhibits a negative correlation with obesity and diabetes [52]. Prior research has showed that a reduction in the prevalence of *Rikenella* has beneficial impacts on enhancing intestinal barrier function, mitigating aging, managing diabetes-related ailments, and reducing inflammation [53]. This research underscored the regulatory potential of EcN-GLP-1 in balancing the intestinal microbial landscape, thereby ameliorating symptoms in DM mice.

In T2DM mice, the application of EcN-GLP-1 prominently decreased blood glucose levels and ameliorated weight gain symptoms (Figure 5). Significantly, we observed a substantial improvement in insulin resistance, a crucial pathological indicator used to assess glucose metabolism efficiency and insulin sensitivity in individuals with T2DM [50]. This improvement highlights the positive impact of EcN-GLP-1 on regulating blood glucose metabolism. Unlike the findings observed in the T1DM mouse model, EcN-GLP-1 exhibited the capability to mitigate pancreatic inflammation by reducing the expressions of MyD88, while the effects on TLR-4 and p-NF-*κ*B/NF-*κ*B were not obvious. Similarly, the impact of EcN-GLP-1 on cellular apoptosis (Bax/Bcl-2 ratio) was also not significant, but the results of immunofluorescence analysis indicated increased insulin production. Furthermore, EcN-GLP-1 contributed to the upkeep of intestinal microbiota equilibrium and demonstrated the potential to diminish lipid accumulation (Figures 5, 6, and 7). Collectively, these findings suggest that EcN-GLP-1 holds promise as an effective approach for both preventing and treating T2DM.

Compared with other oral GLP-1 analogs, one of the prime and unique advantages that EcN-GLP-1 presents lies in its capability for a sustained and continuous release of GLP-1 directly within the intestinal environment. This mechanism has the potential to mimic the natural physiological release pattern more closely, enhancing the overall therapeutic effect. Furthermore, EcN-GLP-1 might confer additional benefits such as enhanced bioavailability, reduced dosage frequency, synergistic effects, and lowered systemic side effects. However, it is imperative to underscore that these theoretical advantages must be validated through meticulous preclinical and rigorous clinical trials. In addition, while this study has provided valuable insights into the potential benefits of EcN-GLP-1 in the context of diabetes management, several limitations warrant consideration: ① The primary focus of this study centered on discerning the effects of EcN-GLP-1 over a 9-week period. However, it is crucial to conduct a thorough assessment of the long-term effects of EcN-GLP-1 treatment. Prolonged and extensive investigations are indispensable to establish the enduring sustainability of the observed benefits. ② While the study delved into alterations in gut microbiota, it omitted exploration of the primary metabolites originating from the probiotics. The intestinal microecology encompasses not just the gut microbiota itself but also a range of metabolites, which include SCFAs. SCFAs, functioning as vital signaling molecules between the gut microbiota and other organs, often mediate the impact of most probiotics on intestinal health. ③ As for the locale of GLP-1 synthesis, theoretical considerations point toward the small intestine as the optimal GI compartment. However, pinpointing the exact segment—be it the duodenum, jejunum, or ileum—would necessitate deeper investigation and fine-tuning to ensure both the optimal functionality and stability of GLP-1 amidst the complex GI environment. Therefore, comprehensive, extended-duration investigations coupled with metabolomics analyses are essential not only to ascertain the durability of observed advantages but also to unravel the intricate metabolic mechanisms underpinning the hypoglycemic effects of EcN-GLP-1.

## 5. Conclusion

To summarize, this study unveils the antidiabetic effects of the extended-release GLP-1 engineered bacterium, EcN-GLP-1, in mammals. These effects encompass an augmentation in the population of islet *β*-cells, attenuation of inflammatory responses and apoptosis, enhancement of liver lipid metabolism, and the normalization of microbial diversity. These findings hold significant implications for informing the development and potential clinical implementation of novel DM therapeutics. Nevertheless, gaining a more profound understanding of the underlying mechanisms is crucial for future research endeavors.

## Data Availability

The datasets presented in this study can be found in online repositories. The names of the repository/repositories and accession number can be found in PRJNA1016258.

## Supporting Information

The regulation of EcN-GLP-1 on the abundance of intestinal beneficial bacteria in T1DM mice and T2DM mice are shown in Supporting Figure 1, which includes the relative abundance of *Alloprevotella*, *Parasutterella*, *Escherichia*, *Bacteroides*, *Helicobacter*, and *Lactobacillus* in T1DM mice, and *Alistipes*, *Clostridium IV*, *Bacteroides*, *Escherichia*, and *Rikenella* in T2DM mice.
