# Supplementary material for: Administration of a Next-Generation Probiotic Escherichia coli Nissle 1917-GLP-1 Alleviates Diabetes in Mice With Type 1 and Type 2 Diabetes
Source: Can J Infect Dis Med Microbiol. 2025 Jan 29;2025:6675676. doi: 10.1155/cjid/6675676 (PMC11824388; doi:10.1155/cjid/6675676)
Supplement: Supporting Information — Additional supporting information can be found online in the Supporting Information section. [file 6675676.f1.pdf]

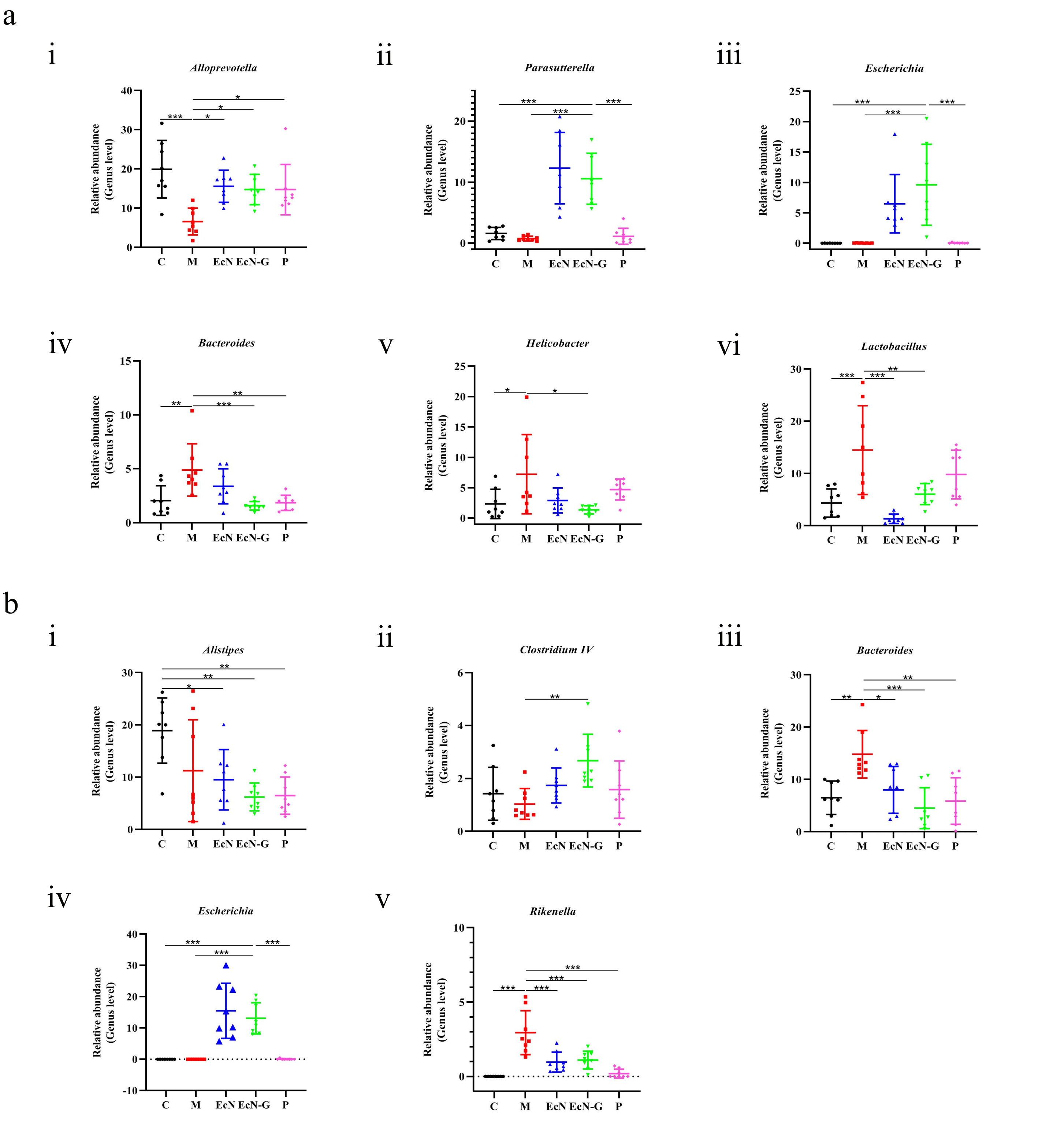

Figure S1 The regulation of EcN-GLP-1 on the abundance of intestinal beneficial bacteria in T1DM mice and T2DM mice. (a) The relative abundance of *Alloprevotella* (i), *Parasutterella* (ii), *Echerichia* (iii), *Bacteroides* (iv), *Helicobacter* (v) and *Lactobacillus* (vi) were analyzed in T1DM mice; (b) The relative abundance of *Alistipes* (i), *Clostridium IV* (ii), *Bacteroides* (iii), *Echerichia* (iv) and *Rikenella* (v) were analyzed in T2DM mice. Data are presented as means ± SD. One way repeated-measures ANOVA with Tukey's test for multiple comparisons; *$p < 0.05$, **$p < 0.01$, ***$p < 0.001$.
